# Tonsillar Cancer with High CD8^+^ T-Cell Infiltration Features Increased Levels of Dendritic Cells and Transcriptional Regulation Associated with an Inflamed Tumor Microenvironment

**DOI:** 10.3390/cancers13215341

**Published:** 2021-10-25

**Authors:** David Gomez Jimenez, Aastha Sobti, David Askmyr, Christina Sakellariou, Sofia Carreira Santos, Sabine Swoboda, Ola Forslund, Lennart Greiff, Malin Lindstedt

**Affiliations:** 1 Department of Immunotechnology, Lund University, 223 81 Lund, Sweden; david.gomez_jimenez@immun.lth.se (D.G.J.); aastha.sobti@immun.lth.se (A.S.); christina.sakellariou@immun.lth.se (C.S.); sofiaccss@gmail.com (S.C.S.); 2 Department of ORL, Head & Neck Surgery, Skåne University Hospital, 221 85 Lund, Sweden; david.askmyr@med.lu.se (D.A.); sabine.swoboda@med.lu.se (S.S.); lennart.greiff@med.lu.se (L.G.); 3 Department of Clinical Sciences, Lund University, 221 85 Lund, Sweden; 4 Department of Microbiology, Lund University, 221 85 Lund, Sweden; ola.forslund@med.lu.se

**Keywords:** tonsillar cancer, human papillomavirus, tumor microenvironment, CD8^+^ T-cells, dendritic cells, macrophages

## Abstract

**Simple Summary:**

Human papilloma virus (HPV) infection is the leading cause of tonsillar cancer (TC), and HPV^+^ TC has a favorable prognosis as compared to HPV^−^ TC. Further, high levels of CD8^+^ T-cells in TC are associated with a favorable prognosis. Increased understanding of the tumor immune microenvironment is needed to identify prognostic biomarkers and novel treatment targets. In this study, we assess the immune profiles of fresh TC biopsies and contralateral healthy tissue against a detailed HPV analysis, including DNA quantification and RNA expression analysis. We demonstrate a correlation between levels of CD8^+^ T-cells and antigen-presenting cells in TC, and we further present differentially expressed biomarkers and signaling pathways in CD8^HIGH^ vs. CD8^LOW^ HPV^+^ TC. Our study thus describes immune phenotypes and the heterogeneity of the immune compartment in HPV^+^ TC. Further studies are warranted to assess if this information can be used on an individual basis to aid in prognosis and treatment decisions.

**Abstract:**

Human papillomavirus (HPV) is the main causal agent of tonsillar cancer (TC) and HPV^+^ TC has a favorable prognosis compared to HPV^−^ disease. In this study, we examined aspects of the tumor microenvironment of TC, focusing on T-cells, dendritic cells (DC), and macrophages. Fresh biopsies of TC and the contralateral healthy tonsil (HT) were obtained from 20 patients, analyzed by multiparameter flow cytometry, and assessed against a detailed HPV-status. Additionally, RNA-sequencing data from 38 TC samples available in the public database, The Cancer Genome Atlas (TCGA), were explored, focusing on the same leukocyte populations. HPV^+^ TC featured increased levels of CD8^+^ T-cells and antigen-presenting cells (cf. HPV^−^ TC and HT, respectively). In HPV^+^ TC, CD8^+^ T-cell frequencies correlated to DC levels independently of tumor stage, HPV 16 copy number, and E7 oncogene expression as well as frequencies of other leukocytes. Similarly, RNA sequencing data were explored by dividing the HPV^+^ TCs according to predefined CD8^+^ T-cell scores in silico. Higher levels of genes expressed by antigen-presenting cells and effector T-cells, such as immune checkpoints and cytokines, were detected in the CD8^HIGH^ HPV^+^ TC samples (cf. CD8^LOW^ HPV^+^ TC). In conclusion, CD8^HIGH^ HPV^+^ TC displays a unique inflammatory profile associated with increased effector T-cell functions and the presence of antigen-presenting cells in the tumor microenvironment. Further studies are warranted to assess if this information can be used on an individual basis to aid in prognosis and treatment decisions.

## 1. Introduction

Tonsillar cancer (TC) is a subset of oropharyngeal cancer (OPC). In addition to tobacco smoking and alcohol abuse, high-risk human papillomavirus (HPV), notably HPV16, is a cause of the disease [1,2]. Indeed, in Sweden, over 70% of TCs are HPV^+^ [3,4]. As for solid cancers in general, aspects of the intralesional immune profile are emerging as potential prognostic tools and treatment targets for TC and OPC. Notably, the survival of patients with HPV^+^ disease may vary depending on whether or not an immune response occurs. For example, patients with detectable levels of antibodies against HPV oncoproteins E6 and E7 in OPC have a more favorable prognosis than those without a humoral immune response [5]. Such observations indicate the necessity to assess the tumor microenvironment of HPV^+^ TC, as the tumor immune phenotypes may be used to improve clinical protocols, e.g., resulting in escalation or de-escalation of treatment.

A series of studies has addressed the immune composition and its heterogeneity in OPC/TC, including aspects of the main leukocyte populations involved in initiating and sustaining antigen-specific cell-mediated immunity, such as dendritic cells (DC), macrophages, and T-cells. As originally suggested by Galon et al. for colorectal cancer [6], Näsman et al. and Nordfors et al. reported that high levels of tumor-infiltrating CD8^+^ T-cells in TC, indicating an “inflamed” phenotype, predicted a favorable outcome [7,8]. Subsequently, Welters et al. detailed the immune compartment in OPC and indicated that HPV-associated disease is rich in tissue-resident effector and memory T-cells [9]. In a previous study, we demonstrated the presence of DCs in TC, i.e., CD123^+^ plasmacytoid DCs (pDC), CD1c^−^ CD141^−^ DCs, as well as CD1c^+^ and CD141^+^ conventional DCs (cDC), and indicated subset-specific immune features [10]. Moreover, Santegoets et al. identified CD163^+^ DCs in HPV^+^ OPC associated with CD4^+^ T-cell abundance [11]. Oguejiofor et al., focusing on OPC in general, reported increased levels of CD68^+^ macrophages as associated with HPV^+^ disease and proposed an association of CD68^+^ PD-L1^+^ cells and survival [12].

Taken together, while detailed and of great interest, the above-mentioned studies [7,8,9,10,11,12] may be hampered by a few aspects. For example, HPV presence and activity, in terms of HPV-DNA levels and transcription of oncogenes E6 and E7, are not always indicated in relation to immune responses recorded. Furthermore, the characterization of TME in OPC is potentially hampered by subsite-specific divergencies. Unlike, for instance, cancer of the soft palate, TC develops in tonsillar crypts [13], which are in close contact with extrafollicular areas where T-cell priming occurs, arguably increasing the availability of tumor-associated antigens to DCs. The TME of TC may thus be best defined as a separate entity, and, arguably, an appropriate control tissue is the contralateral tonsil of the patient in question, as it reduces observations related to inter-individual variability. Finally, there is a technical limitation when defining macrophages using CD68 as a single marker, given the promiscuous expression of CD68 also, e.g., by tonsillar DC populations [14,15].

In this study, we examined the TME specifically of TC, focusing on the presence/status of DCs, macrophages, and T-cells assessed by multiparameter flow cytometry. Notably, we compared TME data between biopsies obtained from the TC lesion and the contralateral healthy tonsil (HT) of the same patient and assessed the results in relation to HPV status. In addition, publicly available RNA sequencing data of whole TC tissue were explored in silico. We demonstrated that HPV^+^ TC lesions feature high and transcriptionally active HPV-DNA levels (cf. contralateral HTs). Furthermore, we showed that CD8^+^ T-cell levels are increased in HPV^+^ TC (cf. HPV^−^ TC as well as HT), and that CD8^HIGH^ HPV^+^ TC features specific characteristics regarding immune cell and gene expression profiles (cf. CD8^LOW^ HPV^+^ TC as well as HT). Moreover, we demonstrated that macrophage and DC levels are high in HPV^+^ TC (cf. HT) and that CD8^+^ T-cell levels correlate with DC abundance, but not to macrophage infiltration, CD4^+^ T-cell presence, or HPV16 E7 DNA CN or E7 mRNA levels.

## 2. Results

### 2.1. HPV Presence in TC and HT

**Figure 1:**
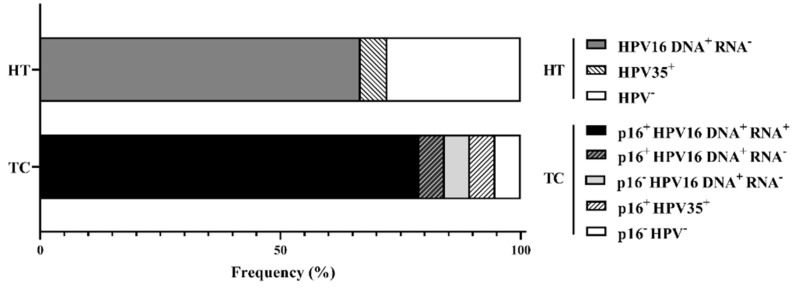
HPV-status of TC and contralateral HT according to p16, HPV E7 DNA, and RNA detection. Tonsillar cancer (TC); healthy tonsil (HT). p16-status (at the time of diagnosis), HPV16 DNA (detection of HPV E7 DNA in fresh biopsies), HPV16 RNA (detection of E7 mRNA normalized over GAPDH levels in fresh biopsies).

The HPV status of TC and contralateral HT was assessed by HPV typing as well as HPV16 E7 DNA and mRNA quantification through PCR (Table 1, Figure 1). Additionally, p16 immunohistochemistry was available from the clinical assessment at diagnosis. HPV DNA was detected in 95% of TC samples and 70% of contralateral HT, with a high prevalence of HPV16 in both tissues (Figure 1). A total of 83% of HPV16 DNA^+^ TC samples had levels of E7 DNA over one copy per cell and detectable levels of HPV16 E7 mRNA (Table 1). In contrast, all HPV16 DNA^+^ HT samples displayed E7 DNA levels below one copy per cell, and E7 mRNA expression was not detected (Table S1). Additionally, two HPV16 DNA^+^ TC cases with viral DNA titers below one copy per cell and undetectable oncogene expression were classified as HPV^−^ in subsequent analyses.

### 2.2. Immune Cell Infiltration and HPV Status

**Figure 2:**
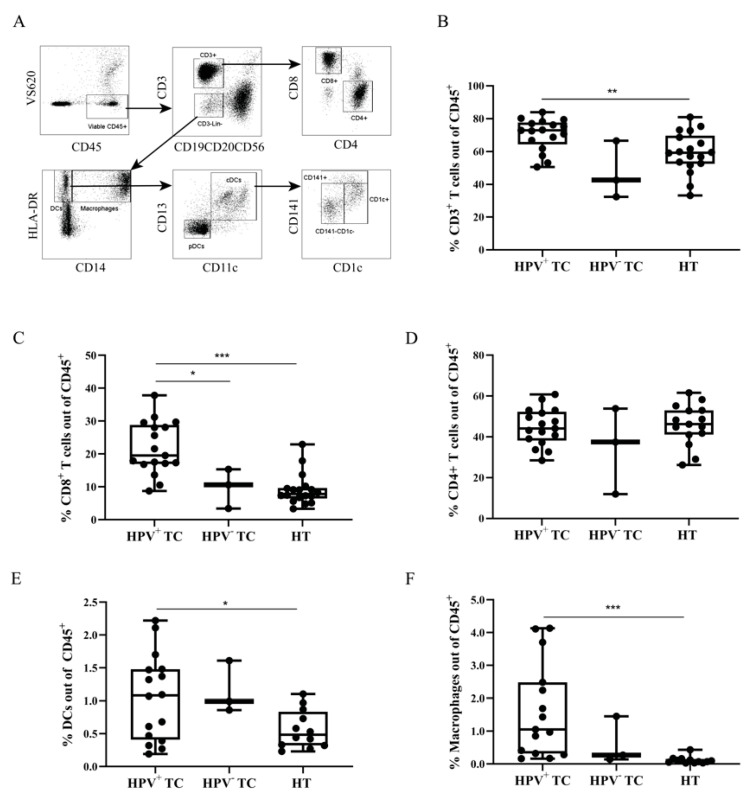
Frequency of infiltrating leukocyte populations in TC and HT. (**A**) Gating strategy: After doublet exclusion on viable cells, leukocytes were gated according to CD45 expression. T-cells were gated as CD3^+^ Lineage^−^ (Lin; CD19, CD20, and CD56) and subdivided into CD8^+^ and CD4^+^ cells. Antigen presenting cells (CD3^−^ Lin^−^HLA-DR^+^) were divided into CD14^−^ DCs and CD14^+^ macrophages. cDCs and pDCs were classified as CD11c^+^ CD13^+^ and CD11c^−^ CD13^−^, respectively. cDCs were further classified as CD141^+^, CD1c^+^, and CD141^−^ CD1c^−^. (**B**–**F**) Box plots comparing frequencies of (**B**) CD3^+^ T-cells, (**C**) CD8^+^ T-cells, (**D**) CD4^+^ T-cells (**E**) DCs, and (**F**) macrophages according to tissue and HPV status. Adjusted *p*-values are shown as * < 0.05, ** < 0.01, *** < 0.005. Dendritic cell (DC); conventional DC (cDC); plasmacytoid DC (pDC); copy number (CN).

Using flow cytometry, the main leukocyte populations involved in initiating and sustaining antigen-specific cell-mediated immunity were identified in TC (Figure 2A), compared with HT, and assessed against HPV16-status. Higher percentages of total CD3^+^ T-cells were detected in HPV^+^ TC (cf. HT) (Figure 2B). Further, the levels of CD8^+^ T-cells were significantly increased in HPV^+^ TC (cf. HPV^−^ TC and HT) (Figure 2C), while CD4^+^ T-cell levels were similar in TC and HT and independent of HPV status (Figure 2D). CD14^+^ macrophages and CD14^−^ DC subsets were identified in the CD3^−^ Lin^−^ HLA-DR^+^ gate (Figure 2A). Higher percentages of total DCs and macrophages were observed in the HPV^+^ TC samples as compared to HT (Figure 2E,F), indicating active recruitment of myeloid cells in the TME. No differences in frequencies of T-cells or APCs were observed between HPV^+^ and HPV^−^ HT (Figure S1).

### 2.3. Variables Correlating with CD8^+^ T-Cell Frequency

**Figure 3:**
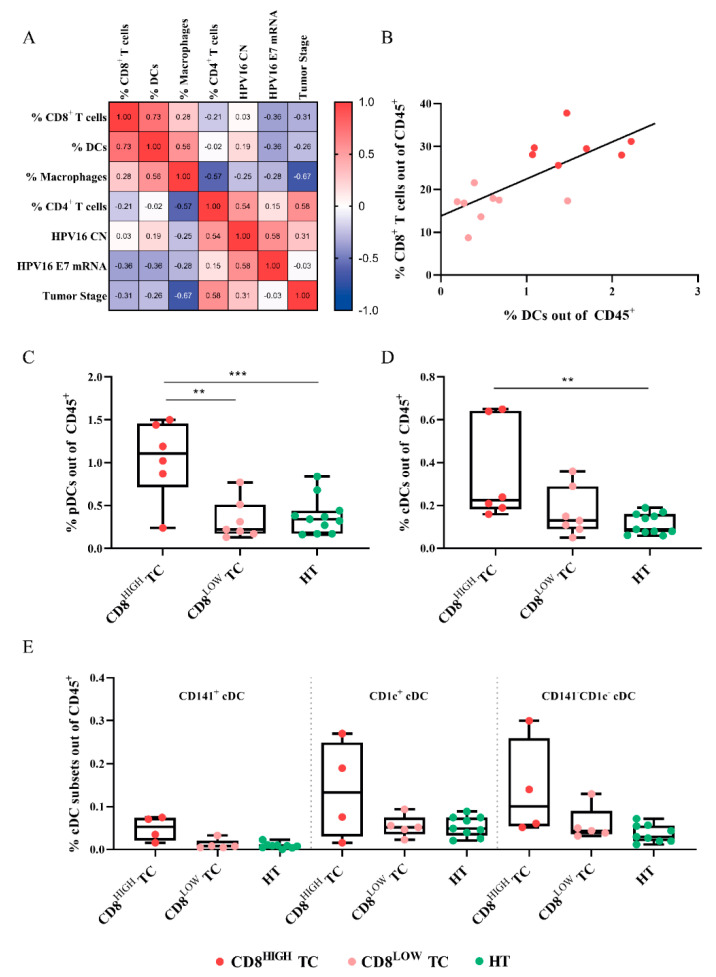
Frequencies of tumor-infiltrating DCs in relation to CD8^+^ T-cells in HPV16^+^ TC. (**A**) Correlation matrix displaying Pearson coefficient (r) between the different variables. Cell abundance is expressed out of CD45^+^ leukocytes. (**B**) Correlation between CD8^+^ T-cell and DC infiltration in HPV^+^ TC. Samples colored in red represent CD8^HIGH^ HPV16^+^ TC, and samples colored in salmon pink represent CD8^LOW^ HPV16^+^ TC. Box plots showing (**C**) pDC, (**D**) cDC, and (**E**) cDC subsets’ relative abundances according to CD8^+^ T-cell infiltration and tissue type. Copy number (CN); dendritic cell (DC); plasmacytoid DC (pDC); conventional DC (cDC). Adjusted *p*-values are shown as ** < 0.01, *** < 0.005.

Multiple correlation analysis was performed to investigate factors associated with CD8^+^ T-cell infiltration in HPV^+^ TC (Figure 3A). Relative abundance of DCs, macrophages, and CD4^+^ T-cells, as well as tumor stage, HPV16 E7 DNA CN, and E7 mRNA levels, were selected as variables. Among these, only the frequency of DCs was correlated with CD8^+^ T-cells (Figure 3B). Subdivision of DCs into pDC and cDC revealed that pDC infiltration was significantly correlated with CD8^+^ T-cell frequency (*p* = 0.0024), whereas cDC frequency was not (*p* = 0.0732) (Figure S2). In order to analyze the distribution of DC subsets according to CD8^+^ T-cell infiltration, HPV^+^ TC were subdivided into CD8^HIGH^ and CD8^LOW^ groups, with respect to the median percentage of CD8^+^ T-cells. Both pDCs and cDCs were more abundant in the CD8^HIGH^ group (cf. HT), and pDCs were also significantly more prevalent in the CD8^HIGH^ group (cf. CD8^LOW^ TC) (Figure 3C,D). Similar trends were observed for the cDCs subsets CD141^+^, CD1c^+^, and CD141^−^ CD1c^−^, but these differences failed to reach statistical significance (Figure 3E).

### 2.4. Immunoprofiling of TC Using TCGA Data

RNAseq data from TCGA were assessed to profile the immune compartment of TC. A two-group comparison revealed 1047 DEGs that separated HPV^+^ and HPV^−^ TC. Highly expressed genes in HPV^+^ TC showed enrichment in T-cell–cell activation and differentiation (cf. HPV^−^ TC), e.g., “T-cell receptor activation’’ as well as “Th1-, Th2-, and Th17-differentiation’’ (Table S2). On the other hand, genes over-expressed in the HPV^−^ TC (cf. HPV^+^ TC) corresponded to intracellular signaling and reassembling of the cellular matrix, e.g., “PI3K-Akt signaling pathway’’, “Focal adhesion’’, and “Extracellular matrix receptor interaction’’ (Table S3).

**Figure 4:**
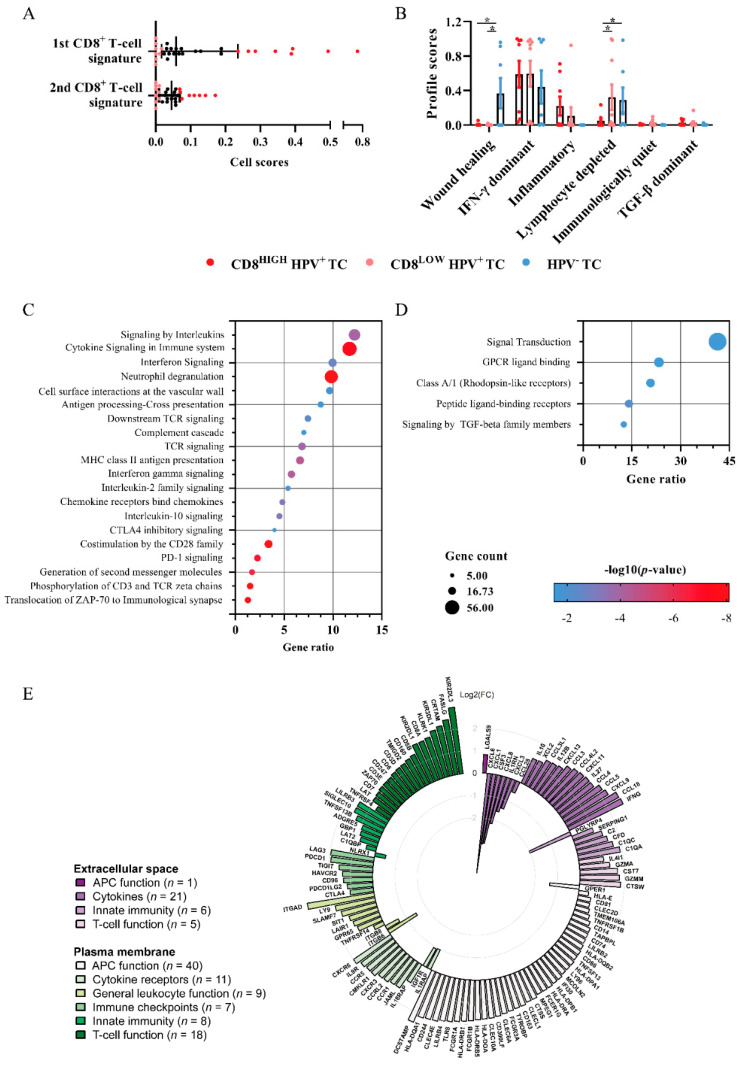
Transcriptomic analysis of whole tissue RNAseq TC datasets from TCGA. (**A**) Scatter plots showing the division of HPV^+^ TC samples based on cell signatures into CD8^HIGH^ and CD8^LOW^HPV^+^ TC [18,19]. (**B**) Profile scores of the groups based on six TCGA immune signature profiles [17]. (**C,D**) Bubble plot displaying REACTOME pathway enrichment in CD8^HIGH^ HPV^+^ and CD8^LOW^ HPV^+^ TC, respectively. Each bubble represents the gene ratios, defined as the ratio of genes in the pathway divided by the total genes obtained. The size corresponds to the number of obtained genes in a pathway and the color with –LOG10 *p*-value. (**E**) Sunburst circos plots showing 126 DEG between CD8^HIGH^ and CD8^LOW^ HPV^+^ TC, associated with extracellular space and plasma membrane. The immune-related genes are distributed according to the FC values. Differentially expressed genes (DEGs); fold change (FC); The Cancer Genome Atlas (TCGA). Adjusted *p*-values are shown as * < 0.05.

The CD8^+^ T-cell signatures defined by Puram et al. and Newman et al. via CIBERSORTX [16] were used to divide the HPV^+^ TC samples into CD8^HIGH^ (*n* = 8) and CD8^LOW^ (*n* = 9) groups (Figure 4A). The transcriptional data from the CD8^HIGH^ HPV^+^, CD8^LOW^ HPV^+^, and HPV^−^ TC groups were analyzed using a previously described immunogenomics pipeline [17], with signatures defining six immune subtypes in cancer, i.e., wound healing, IFN-γ dominant, inflammatory, lymphocyte depleted, immunologically quiet, and TGF-β dominant. While most samples were related to a heightened IFN-γ signature, the HPV^−^ TC group demonstrated a significantly higher wound healing profile (cf. CD8^LOW^ and CD8^HIGH^ HPV^+^ TC). Furthermore, the CD8^HIGH^ HPV^+^ TC group presented a significantly lower profile score for lymphocyte depletion (cf. CD8^LOW^ HPV^+^ and HPV^−^ TC) (Figure 4B).

The two-group comparison between CD8^HIGH^ HPV^+^ and CD8^LOW^ HPV^+^ TC samples revealed 1008 DEGs. Moreover, pathway enrichment analysis revealed a clear profile of immune activation with higher expression of genes in the CD8^HIGH^ HPV^+^ group involved in pathways such as “Signaling by interleukins’’, “Cytokine signaling in immune system’’, “Interferon signaling’’, “Neutrophil degranulation’’, “Antigen processing-cross presentation’’, “MHC class II antigen presentation’’, “CTLA4 inhibitory signaling’’, and “PD-1 signaling’’ (Figure 4C). On the other hand, pathways enriched in the CD8^LOW^ HPV^+^ TC samples included “Signaling by TGF-beta family members”, “Peptide ligand-binding receptors”, and “Signal Transduction”. (Figure 4D). Furthermore, immune-related DEGs between CD8^HIGH^ and CD8^LOW^ were divided for transcripts coding extracellular and plasma membrane proteins (Figure 4E). CD8^HIGH^ HPV^+^ TC samples displayed higher expression of genes involved in T-cell function (e.g., granzyme genes, FASLG, and TNFRSF4), APC functions (e.g., C-type lectin, Fc receptors, and HLA class II genes CD163 and CD14), cytokines (e.g., IFNG, IL12B, IL10, CXCL9, CXCR3, CCL3–5, and XCL2), cytokine receptors (CCR1, CCR5, CXCR3, and CXCR6), immune checkpoints (e.g., PDCD1 (PD1) and PDCDLG2 (PDL2)), innate immune response (e.g., C1QA, C1QC, and C2) and general leukocyte functions (e.g., ITGAD, LY9, and SLAMF7).

## 3. Discussion

In this study, we investigate aspects of the TME of TC, focusing on T-cells, DCs, and macrophages. Fresh tumor material is analyzed, compared to the contralateral HT, and assessed against HPV status. Taken together, we reveal a significant correlation between CD8^+^ T-cells and DCs within the TME of HPV^+^ TC, irrespective of other immune populations and HPV status. We indicate that CD8^HIGH^ HPV^+^ TC patients feature an increased expression of genes related to antigen presentation, T-cell effector profile, and immune checkpoints. Our observations may be relevant for the identification of treatment targets pertinent to TC, as well as for prognostic markers that can potentially permit individualized treatment.

HPV is a distinctive factor associated with a good clinical outcome for TC, arguably because it facilitates an antigen-specific inflammatory response. In this study, we observed that while HPV-DNA was present in HPV^+^ TC lesions as well as in contralateral HTs, the HPV-DNA load was much greater in the former. Furthermore, we observed that a combination of HPV16 E7 DNA and mRNA separated TC lesions (these features present) from HTs (absent). The presence of HPV beyond the cancer lesion has previously been highlighted [20], but the implication of HPV DNA in healthy tissues is not fully understood. We did not detect any significant differences in frequencies of cell populations in HPV DNA positive and negative HT samples. Our observations suggest that transcriptional activity of HPV, likely leading to antigen production, facilitates TC, but that the mere presence of HPV-DNA is trivial in this context.

In addition to HPV status and activity, a high degree of intralesional CD8^+^ T-cell infiltration is a favorable prognostic factor in TC [7,8]. Using the TCGA dataset, we could indeed confirm a greater survival for patients with CD8^HIGH^ HPV^+^ cf. HPV^−^ TC lesions (Figure S3). A trend towards higher survival was also observed for patients with CD8^HIGH^ HPV^+^ cf. CD8^LOW^ HPV^+^ TC-lesion (not statistically significant). In this study, data from flow cytometric and transcriptomic analyses support HPV^+^ TC as predominantly rich in CD8^+^ T-cells, suggesting an “inflamed” or “immune excluded” rather than a “desert” phenotype [21]. However, reflecting inter-individual variation, HPV^+^ TCs displayed a wide spectrum of CD8^+^ T-cell levels. Further studies are required to investigate the aspects associated with the heterogeneous infiltration of CD8^+^ T-cells in HPV^+^ TC, potentially to identify patients who pose a particular therapeutic challenge.

This study characterizes changes in the TME composition that underlie the heterogeneous distribution of CD8^+^ T-cells in HPV^+^ TC patients. CD8^HIGH^ HPV^+^ TC patients feature high levels of DCs and macrophages, indicative of an active recruitment of APCs into the TME. We also demonstrate that the frequency of CD8^+^ T-cells correlates predominantly with DC abundance, specifically with pDCs, rather than with macrophage infiltration, CD4^+^ T-cell presence, or aspects of HPV-status. Interestingly, increased levels of pDCs were seen in the CD8^HIGH^ HPV^+^ TC group (cf. CD8^LOW^ HPV^+^ TC and HT). The precise role of pDCs in tumor immunity is not clear; however, a few studies have correlated the presence of pDCs within the TME with poor prognosis [22,23]. The role of pDCs in viral immunity is better understood. pDCs are well-established type I IFN producers in response to viral infection, such as HPV, and in turn, type I IFNs can induce cDC maturation and CD8+ T-cell expansion [10]. Additionally, a comparison of pDC transcriptomic profiles between TC and HT has been associated with type-I IFN response in TC [10]. Therefore, the increased frequency of pDCs within CD8^HIGH^ HPV^+^ TC patients might be associated with potential increases in type I IFN activity.

We observed an increasing trend of cDC subsets in CD8^HIGH^ HPV^+^ TC (cf. CD8^LOW^ HPV^+^ TC and HT), which, however, did not reach statistical significance. Even though the role of CD141^+^ DCs is key to initiate CD8^+^ T-cell responses through cross-presentation of antigen, their contribution to CD8^+^ T-cell frequency is less evident, especially considering their low abundance [24]. In contrast, CD1c^+^ DCs contribute to cytokine production via secretion of IL-12 and IL-10, which displays their ability to trigger inflammation or tolerance in a TME-dependent manner [10,24]. Intratumoral DC activity may influence CD8^+^ T-cell levels both through priming and subsequent clonal expansion of T-cells at HPV-antigen exposure and through secretion of chemokines inducing the recruitment of CD8^+^ T-cells from the surrounding lymphoid tissue. Taken together, our data indicate that CD8^+^ T-cells levels correlate with overall DC abundance, reflecting the complementary functions of DC subsets.

In order to assess transcriptomic changes in the TME associated with CD8^+^ T-cell infiltration, whole tissue RNAseq datasets of HPV^+^ TCs from the TCGA cohort were compared according to CD8 scores. Patients with high CD8 scores displayed an increase in genes involved in antigen uptake, C-type lectins and Toll-like receptors, and antigen degradation/loading on MHC molecules (e.g., CTSS, IFI30, and TAPBPL). Together, these observations are indicative of active antigen uptake and processing in the TME of HPV^+^ TC with a high level of CD8^+^ T-cells. Additionally, we observed genes involved in antigen presentation (HLA type II molecules), co-stimulation (CD86), and cytokines (IL-12ß, IL-10, and IL-27). Remarkably, among the genes related to antigen presentation, we observed canonical CD1c^+^ DC marker Clec10a, as well as CD163 and CD14 markers, shared between the newly characterized CD163^+^ DC subset and macrophages [11,25]. These observations, along with the absence of B-cell-specific markers, suggest an enrichment of myeloid APCs in the TME of CD8^HIGH^ HPV^+^ TC. However, whole tissue RNAseq cannot delineate the activity of single cells, and a more detailed analysis of myeloid APC is required to elucidate their specific role.

Immunophenotype analysis by signature scores highlights that CD8^LOW^ HPV^+^ and HPV^−^ TC display lymphocyte depletion. In HPV^−^ TC, lymphocyte depletion can be associated with wound healing response and extracellular matrix deposition, leading to immune-exclusion [26]. Conversely, we report that lymphocyte depletion in CD8^LOW^ HPV^+^ TC may represent defective lymphocyte chemotaxis into the tumor. Accordingly, the DEGs upregulated in CD8^HIGH^ HPV^+^ TC (cf. CD8^LOW^ HPV^+^ TC) suggest that intra-tumoral leukocyte chemotaxis encompasses three axes: CCL3/5-CCR5, CXCL9/11-CXCR3, and XCL2. These chemotactic pathways have been reported in the context of APC and T-cell cross-talk [27,28,29,30]. Second, production of IFNγ, by activated CD8^+^ in response to viral particles, stimulates CXCL9/11 expression by a variety of stromal cells, which induce migration of CXCR3^+^ pDCs and T-cells [31]. In fact, upregulation of CXCR3 gene expression by pDCs has been shown in the context of HPV^+^ TC [10]. Validation of the identified pathways and DEGs in CD8^HIGH^ and CD8^LOW^ HPV^+^ TC was not pursued in this study, which is a limitation. Further analyses of, e.g., quantification of identified secreted and cell surface antigens, and identification of the cell types producing these, are thus warranted to fully decipher the TME dynamics in TC.

Constant exposure to HPV viral antigens is reported to contribute to T-cell exhaustion by inducing upregulation of immune checkpoint molecules, thus impairing T-cell activity [32]. In our study, upregulated genes in the CD8^HIGH^ HPV^+^ TC subset (cf. CD8^LOW^ HPV^+^ TC) included PD1, PD-L2, TIGIT, LAG3, CTLA4, and HAVCR2/TIM3, suggesting an exhausted profile [33]. In this context, it was recently reported that activated effector T-cells preferentially expressed immune checkpoint-molecules in OPC as compared to other T-cell populations [34]. However, immune-checkpoint blockade can also result in a treatment response not only through re-activation of pre-existent memory T-cell clones but also by increasing antigen presentation and the consequent generation of a more diverse T-cell response. We hypothesize that patients with high levels of intra-tumoral CD8^+^ T-cells could benefit from immune-checkpoint therapy [35]. Accordingly, we observed increased expression of IFNG-related genes in the CD8^HIGH^ HPV^+^ subset, e.g., CCL5, CXCL9/13, CXCR6, and HLA-DRA, which contribute to a pan-cancer signature suggested to predict an optimum response to anti-PD1 immune checkpoint-therapy [36]. In contrast, TC, with lower levels of CD8^+^ T-cells, may benefit from strategies aiming to increase intra-tumoral antigen presentation. Targeted antigen delivery in vivo, administration of Flt3-LG, or DC vaccination comprise some examples of immunotherapies pursuing increased antigen presentation [37]. Arguably, such immune profiles may be used as prognostic tools to adjust treatments on an individual basis, yet further studies are warranted to examine such possibilities.

## 4. Materials and Methods

### 4.1. Patient Cohort and Sample Collection

The study was approved by the Swedish Ethical Review Authority (ref. no. 2017/580), and written informed consent was obtained from all participating patients. Paired biopsies from TC lesions and contralateral HTs were obtained from 20 patients at the time of diagnosis (Table 1). The biopsies were cut into two parts; the first was stored in RNAlater (Thermo Fisher Scientific, Waltham, MA, USA) and further processed for nucleic acid extraction. The second part was mechanically digested, suspended in RPMI 1640 medium (Thermo Fisher Scientific), incubated for 30 min at 37 °C, and filtered through a 70 µm cell strainer (BD Biosciences, San Jose, CA, USA).

### 4.2. DNA and mRNA Extraction

Each sample was transferred to a 500 µL solution of 4 M guanidinium thiocyanate, 22 mM sodium citrate, 5% sarcosyl, and 1% mercaptoethanol (Sigma-Aldrich, St. Louis, MO, USA) and incubated at room temperature overnight. Extraction of nucleic acids was performed with a Total NA-kit using MagNA Pure LC (Roche, Basel, Switzerland), while mRNA extraction was performed using an Oligotex Direct mRNA Mini Kit (Qiagen, Hilden, Germany). The mRNA was eluted by adding 45 µL of Oligotex elution buffer (70 °C) to the column, followed by 1 min centrifugation at max speed. Purified DNA and RNA were stored at −20 °C and −80 °C, respectively.

### 4.3. HPV Typing, Copy Number, and Viral Gene Expression

Identification of 40 HPV types was carried out by a modified general primer PCR, amplifying a segment of the HPV L1 gene, followed by Luminex analysis (Thermo Fisher Scientific) [38,39]. Viral HPV16 DNA copy number (CN), or the number of viral genomes per cell, was determined as the number of HPV16 E7 DNA copies normalized to the number of β-globin DNA. The HPV16 E7 gene and the human β-globin gene were quantified by two separate real-time PCR tests as described previously [40]. HPV16 E7 oncogene expression was calculated as the fraction of HPV16 E7 mRNA per GAPDH mRNA. Quantitative PCR of HPV16 E7 mRNA was analyzed in triplicate and performed as previously described [40]. Quantitative PCR of GAPDH mRNA was analyzed in triplicate (Supplementary materials and methods).

### 4.4. Flow Cytometry

The characterization and frequency assessment of immune cell populations was performed using a panel of commercially available antibodies (Table 2). Briefly, cells were blocked with ChromPure mouse IgG whole molecule (Jackson ImmunoResearch, West Grove, PA) and incubated for 30 min at 4 °C with the antibody panel and Fixable viability stain 620 (BD Biosciences). Cells were washed and assessed using FACSAria IIu (BD Biosciences), and acquired events were further analyzed using FlowJo Software (FlowJo LLC, Ashland, OR). Fluorescence minus one (FMO) experiments were run to ensure proper gating. After doublets exclusion on viable cells, leukocytes were gated according to CD45 intensity. CD3^+^ T-cells were divided into CD8^+^ and CD4^+^ T-cells, and antigen-presenting cells (APC) were identified as CD3^−^ Lin (CD19, CD20, CD56)^−^ HLA-DR^+^ cells and further subdivided into CD14^−^ DCs and CD14^+^ macrophages. cDCs and pDCs were identified as CD11c^+^ CD13^+^ and CD11c^−^ CD13^−^, respectively. pDC identity was confirmed by including CD123 in subsequent staining, with 90% overlap between CD11c^−^ CD13^−^ and CD123^+^ CD11c^−^ CD13^−^ populations (data not shown). cDCs were subclassified as CD141^+^ CD1c^+^, CD141^−^ CD1c^+^, and CD141^−^ CD1c, accounting for the total number of cDCs. The frequency of an individual population was calculated in relation to the tissue infiltrating CD45^+^ leukocytes using FlowJo software. Only samples with more than 100 events included in the DC gates were considered for DC subpopulations analysis. Statistical analysis and plotting were conducted in GraphPad Prism 9.0 (GraphPad Software, La Jolla, CA, USA). Differences in mean percentages of leukocyte populations were assessed by either unpaired Welch’s *t*-test or Mann–Whitney test, after evaluating normality using the Shapiro–Wilk test. *p*-values < 0.05 were considered significant. A correlation matrix was built using Pearson’s correlation coefficients to evaluate variables related to the frequency of CD8^+^ T-cells. Linear correlations were considered significant when r > 0.7 and *p*-values < 0.05. Further grouping of HPV16^+^ TC samples into CD8^HIGH^ (3rd and 4th quadrants) and CD8^LOW^ (1st and 2nd quadrants) was performed based on their frequency of CD8^+^ T-cells.

### 4.5. TC Transcriptomic Data Acquisition and Analysis

Clinical and transcriptomic data for 38 TC samples were retrieved from the GDC portal repository of The Cancer Genome Atlas (TCGA) [41]. The information for uncategorized samples, lacking data on HPV status, was retrieved from a previous study in which RNA-sequence libraries were aligned to high-risk HPV subtype genomes [42], resulting in 7 HPV^−^ samples and 31 HPV16^+^ samples in the present cohort. The RNA-Seq data were normalized using edgeR version 3.30.3 [43,44].

Differentially expressed genes (DEGs) between HPV^+^ and HPV^−^ TC were identified by two-tailed *t*-test (*p*-value < 0.01, FC > 2, variance > 0.2) using Qlucore Omics Explorer 3.6 (Qlucore, Lund, Sweden). The obtained DEGs were enriched for KEGG pathways in Stringdb, available on Cytoscape [45].

The HPV^+^ TC group was further divided into CD8^HIGH^ and CD8^LOW^ subgroups using two previously defined CD8 T-cell signatures [18,19]. After batch correction, gene expression values were determined for the two signature profiles using CIBERSORTX [16]. Samples with correlation > 50% and *p*-value < 0.05 were further considered for analysis (*n* = 27). To avoid bias from one signature, only those samples overlapping in both signatures were termed as CD8^HIGH^ (4th quadrant, *n* = 8) and CD8^LOW^ (1st quadrant, *n* = 9). The remaining TC samples (2nd and 3rd quadrants) were removed from further analyses. Differences among the TC groups were further assessed using six pan-cancer immune subtype signatures [17] and compared using a 2-way ANOVA Tukey test. DEGs between CD8^HIGH^ and CD8^LOW^ HPV^+^ TC were estimated by two-group comparison using a Mann–Whitney test (*p*-value < 0.05, fold change =1.5). DEGs obtained were then subjected to REACTOME pathway enrichment using Stringdb, available on Cytoscape [45]. Bar plots and bubble plots were generated with GraphPad Prism. DEGs between CD8^HIGH^ and CD8^LOW^ HPV^+^ TC were further explored using Gene ontology (GO:0002376 and GO:0006955) and Ensembl annotations, accessed through the biomaRt package [46] (version 2.46.2). Circos plots were created using the Circlize package (version 0.4.12) in R [47]. Survival analysis was performed on the CD8^HIGH^ and CD8^LOW^ HPV^+^, as well HPV^−^, TC patient groups using Kaplan–Meier analysis and log-rank (Mantel–Cox) test.

## 5. Conclusions

In conclusion, we evaluated the immune profiles of fresh TC and contralateral HT samples and demonstrated an increase in CD8^+^ T-cells levels in HPV+ TC cf. HPV^−^ TC and HT. Furthermore, we showed that CD8^+^ T-cells correlated with DC frequency in HPV^+^ TC, and indicated genes associated with an inflamed profile in HPV^+^ TC with high predicted CD8^+^ T-cell infiltration. This subset of patients displayed an increase of antigen presentation, IFN-γ responses, immune-checkpoint regulation, and chemotactic activity-related genes. Further studies are warranted to assess if this information can be used to identify treatment targets and, on an individual basis, for prognostic information and treatment selection.

## Figures and Tables

**Table 1:** Patient characteristics and HPV status in TC.

ID	Age	Sex	Tumor Stage	p16 Status	HPV Type	HPV16/cell (CN)	HPV16 E7/GAPDH mRNA	HPV Annotation
TC01	57	F	I	+	35	NA	NA	HPV^+^
TC02	49	M	I	+	16	33.9	3.5	HPV^+^
TC03	61	F	II	+	16	<1	-	HPV^−^
TC04	60	F	I	+	16	1.3	0.2	HPV^+^
TC05	71	F	II	-	-	-	-	HPV^−^
TC06	57	F	III	+	16	4.4	0.7	HPV^+^
TC07	74	M	II	+	16	35.0	2.6	HPV^+^
TC08	60	M	I	+	16	<1	-	HPV^−^
TC09	53	M	I	+	16	11.0	0.7	HPV^+^
TC10	66	M	III	+	16	772.4	1.2	HPV^+^
TC11	59	F	II	+	16	904.4	11.0	HPV^+^
TC12	61	M	I	+	16	140.0	1.0	HPV^+^
TC13	59	M	III	+	16	57.6	4.8	HPV^+^
TC14	63	M	I	+	16	14.0	4.1	HPV^+^
TC15	61	M	III	+	16	178.1	<0.1	HPV^+^
TC16	69	M	II	+	16	2.8	0.5	HPV^+^
TC17	59	M	II	+	16	67.4	0.3	HPV^+^
TC18	61	F	I	+	16	30.4	0.1	HPV^+^
TC19	62	M	II	+	16	7.4	0.1	HPV^+^
TC20	72	M	II	+	16	3.3	0.6	HPV^+^

Copy number [(CN)]; not applicable (NA); negative (-).

**Table 2:** Antibody panel employed for leukocyte quantification.

Antibody	Clone	Supplier
CD45–APC-H7	2D1	BD Biosciences
CD45–FITC	2D1	BD Biosciences
CD19–PerCP-Cy5.5	HIB19	BD Biosciences
CD20–PerCP-Cy5.5	2H7	BD Biosciences
CD56–PerCP-Cy5.5	B159	BD Biosciences
CD3–BV786	SK7	BD Biosciences
CD4–BV510	SK3	BD Biosciences
CD8–PE-Cy7	RPA-T8	BD Biosciences
CD14–APC	Tuk4	Miltenyi Biotec
HLA-DR–BV711	G46-6	BD Biosciences
CD11c–BV421	B-ly6	BD Biosciences
CD13–PE	WM15	BD Biosciences
CD1c–AF700	L161	Biolegend
CD141–BV605	1A4	BD Biosciences

## Data Availability

The transcriptomics data for tonsillar cancer was obtained from the TCGA repository on 31 January 2019. (https://www.cancer.gov/tcga).

## Supplementary Materials

The following are available online at www.mdpi.com/2072-6694/13/21/5341/s1, Figure S1: Frequencies of infiltrating leukocytes population in TC and HT, Figure S2: Correlation of DC subsets with CD8+ T-cells, Figure S3. Differences in survival of the CD8^HIGH^ and CD8^LOW^ HPV+, as well HPV-, TC patients, using the TCGA data, Table S1: Patient characteristics and HPV status in TC and contralateral HT biopsies, Table S2: KEGG pathways associated with highly expressed DEGs in HPV^+^ TC, Table S3: KEGG pathways associated with highly expressed DEGs in HPV^−^ TC, and Supplementary material and methods.
