# Supplementary material for: Tonsillar Cancer with High CD8+ T-Cell Infiltration Features Increased Levels of Dendritic Cells and Transcriptional Regulation Associated with an Inflamed Tumor Microenvironment
Source: Cancers (Basel). 2021 Oct 25;13(21):5341. doi: 10.3390/cancers13215341 (PMC8582523; doi:10.3390/cancers13215341)
Supplement: Supplementary file 1 [file cancers-13-05341-s001.zip › cancers-1380941-supplementary.pdf]

Supplementary material

Supplementary figures

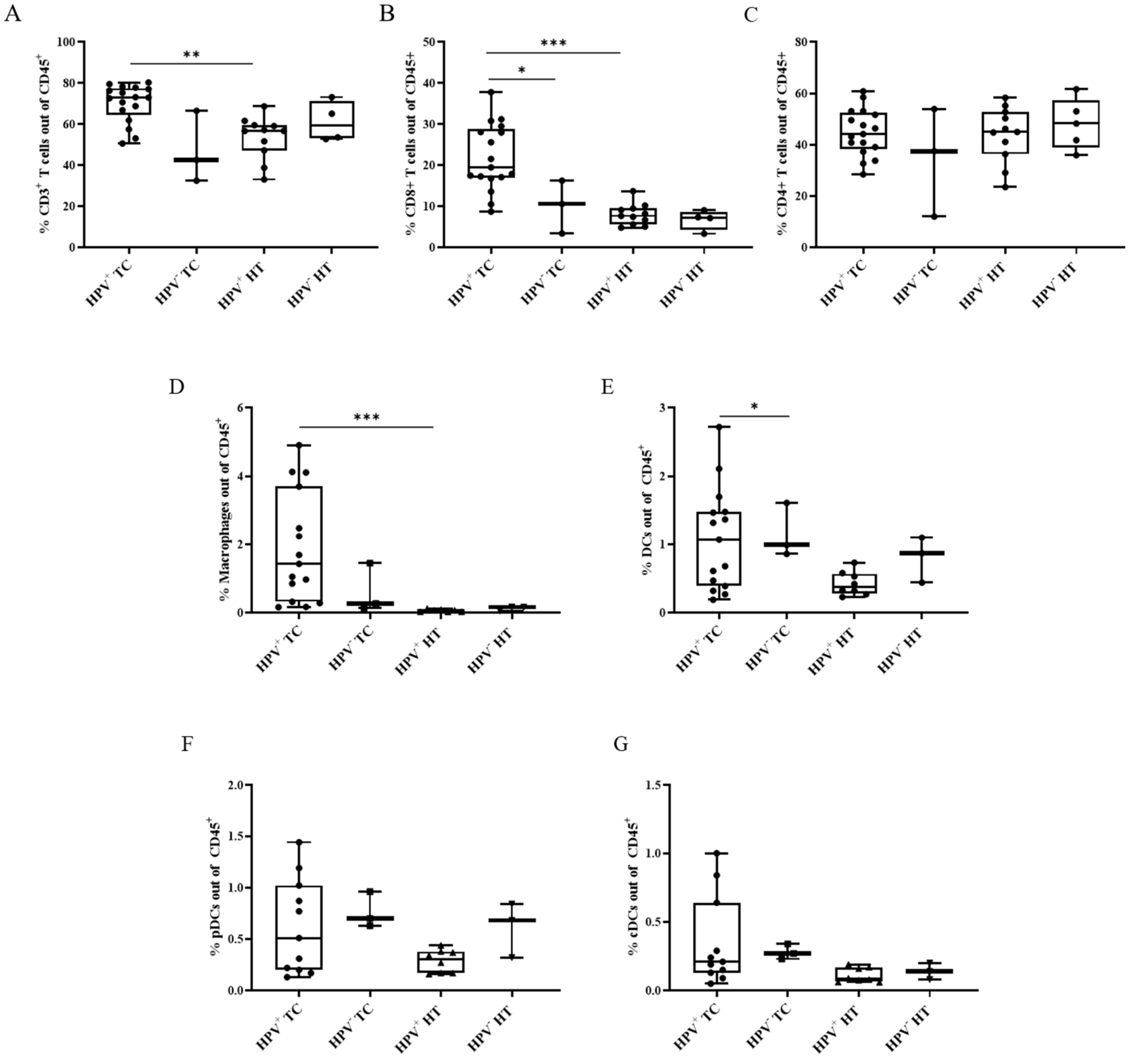

Figure S1. Frequencies of infiltrating leukocytes population in TC and HT. Box plots comparing frequencies of leukocyte populations (**A**) CD3+ T-cells, (**B**) CD8+ T-cells, (**C**) CD4+ T-cells, (**D**) DCs, (**E**) Macrophages, (**F**) pDCs and (**G**) cDCs, according to tissue and HPV status in TC and HT. Adjusted p-values are shown as *< 0.05, **< 0.01, ***<0.001. Tonsillar cancer (TC); Healthy tonsil (HT).

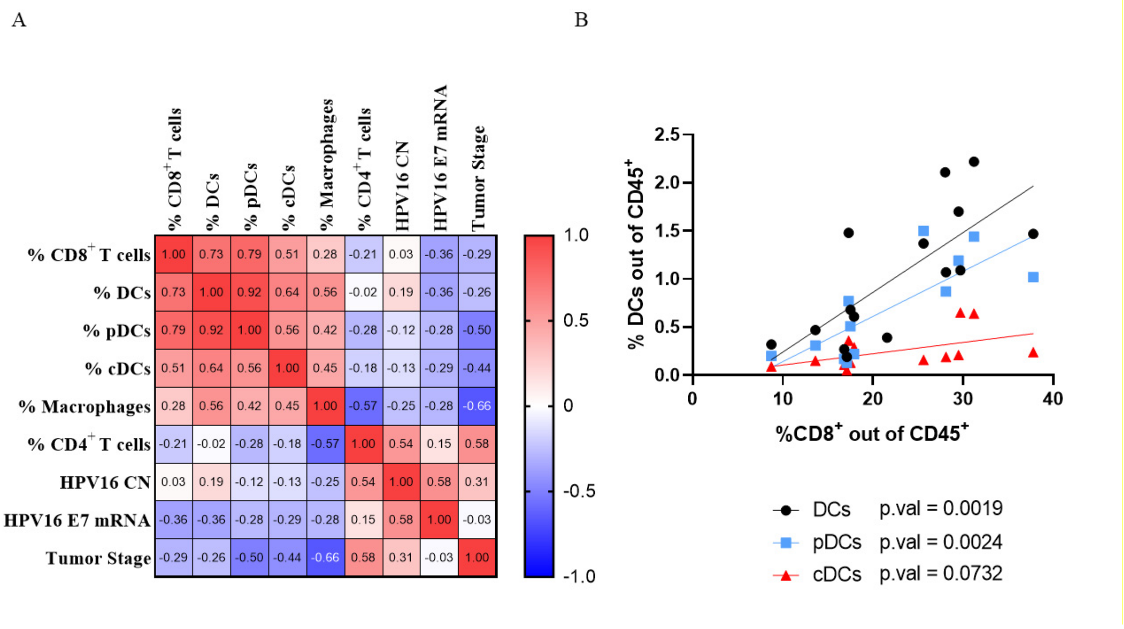

Figure S2. Correlation of DC subsets with CD8+ T-cells. (A) Correlation matrix displaying Pearson's coefficient (r) between the different variables. Cell abundance is expressed out of $CD45^+$ leukocytes. (**B**) Correlation between $CD8^+$ T-cell and DC, pDC, and cDC infiltration, respectively, in HPV^+ TC. Dendritic cell (DC); Plasmacytoid DC (pDC); cDC; Conventional DC (cDC).

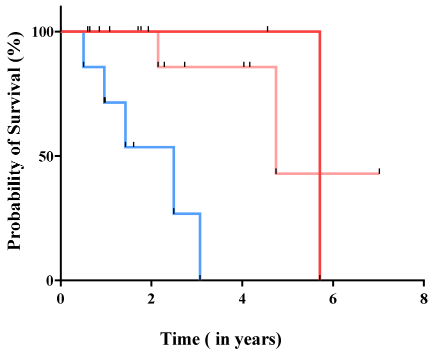

Figure S3. Differences in survival of the $CD8^{HIGH}$ and $CD8^{LOW}$ HPV^+, as well HPV^-, TC patients, using the TCGA data. Kaplan-Meier graphs according to the groups $CD8^{HIGH}$ HPV^+ TC (n=8, red), $CD8^{LOW}$ HPV^+ TC (n=9, salmon, as well HPV^- TC (n=7, blue). A significantly higher survival of $CD8^{HIGH}$ HPV^+ TC patients is observed compared to those with HPV^- TC (p-value<0.0023, Mantel-Cox test).

Supplementary tables

Table S1. Patient characteristics and HPV status in TC and contralateral HT biopsies.

		TC			HT		
**ID**	**p16 status**	**HPV type**	**HPV16 / cell (CN)**	**HPV16 E7/ GAPDH mRNA**	**HPV type**	**HPV16 / cell (CN)**	**HPV16 E7 / GAPDH mRNA**
Patient 01	+	35	NA	NA	35	NA	NA

Patient 02	+	16	33.9	3.8	-	-	-
Patient 03	+	16	7.14 x 10^{-5}	-	-	-	-
Patient 04	+	16	1.3	0.2	-	-	-
Patient 05	-	-	-	-	-	-	-
Patient 06	+	16	4.4	0.7	16	1.8 x 10^{-4}	-
Patient 07	+	16	35.0	2.6	16	BDL	-
Patient 08	+	16	3.0 x 10^{-7}	-	16	5.6 x 10^{-5}	-
Patient 09	+	16	11.0	0.7	16	1.3 x 10^{-4}	-
Patient 10	+	16	772.4	1.2	16	4.1 x 10^{-3}	-
Patient 11	+	16	904.4	11.0	16	2.4 x 10^{-3}	-
Patient 12	+	16	140.0	1.0	16	1.6 x 10^{-3}	-
Patient 13	+	16	57.6	4.8	16	5.7 x 10^{-4}	-
Patient 14	+	16	14.0	4.1	16	5.0 x 10^{-1}	-
Patient 15	+	16	178.1	4.1 x 10^{-4}	16	3.0 x 10^{-4}	-
Patient 16	+	16	2.8	0.5	16	4.9 x 10^{-5}	-
Patient 17	+	16	67.4	0.2	16	BDL	-
Patient 18	+	16	23.3	0.1	16	BDL	-
Patient 19	+	16	3.8	0.1	16	1.1 x 10^{-3}	-
Patient 20	+	16	3.3	0.6	16	3.0 x 10^{-3}	-

Not applicable (NA); Negative (-); Below detection limit (BDL).

Table S2. KEGG pathways associated with highly expressed DEGs in HPV^{+} TC.

Term description	Observed gene count	Background gene count	False discovery rate	Matching proteins in network (labels)
Primary immunodeficiency	12	37	2.6 x10^{-5}	CD19, CD3D, CD3E, CD40LG, CD79A, CD8A, CD8B, CIITA, JAK3, TNFRSF13B, TNFRSF13C, ZAP70
T cell receptor signaling pathway	14	99	6.0 x 10^{-4}	CD247, CD28, CD3D, CD3E, CD3G, CD40LG, CD8A, CD8B, GRAP2, ITK, PDCD1, PIK3R3, RASGRP1, ZAP70
Hematopoietic cell lineage	13	94	1.1 x 10^{-3}	CD19, CD2, CD3D, CD3E, CD3G, CD5, CD8A, CD8B, CR2, HLA-DMB, HLA-DOA, IL7, MS4A1
Th1 and Th2 cell differentiation	12	88	2.0 x 10^{-3}	CD247, CD3D, CD3E, CD3G, HLA-DMB, HLA-DOA, IL12A, IL12RB1, IL2RB, JAK3, TBX21, ZAP70
Cell adhesion molecules (CAMs)	15	139	2.1 x 10^{-3}	CD2, CD226, CD28, CD40LG, CD6, CD8A, CD8B, CLDN7, HLA-DMB, HLA-DOA, ICAM3, ITGAL, NLGN3, PDCD1, TIGIT
Th17 cell differentiation	12	102	4.8 x 10^{-3}	CD247, CD3D, CD3E, CD3G, FOXP3, HLA-DMB, HLA-DOA, IL12RB1, IL2RB, JAK3, TBX21, ZAP70
Intestinal immune network for IgA production	7	44	2.2 x 10^{-2}	CCL28, CD28, CD40LG, HLA-DMB, HLA-DOA, TNFRSF13B, TNFRSF13C

Differentially expressed genes (DEGs); Kyoto Encyclopaedia of Genes and Genomes (KEGG).

Table S3. KEGG pathways associated with highly expressed DEGs in HPV^{-} TC.

Term description	Observed gene count	Background gene count	False discovery rate	Matching proteins in network (labels)
PI3K-Akt signaling pathway	20	348	7.8 x 10^{-4}	AREG, CCND1, CDK6, COL4A6, COL6A1, EPO, GNGT1, IRS1, ITGA3, ITGA5, ITGA6, LAMA3, LAMC2, LPAR3, NGF, PDGFB, PGF, PPP2R2C, TNC, VEGFC
Focal adhesion	15	197	7.8 x 10^{-4}	ACTN1, CAV1, CCND1, COL4A6, COL6A1, ITGA3, ITGA5, ITGA6, LAMA3, LAMC2, PARVB, PDGFB, PGF, TNC, VEGFC
MicroRNAs in cancer	11	149	6.4 x 10^{-3}	CCND1, CDK6, FSCN1, HMGA2, IRS1, ITGA5, PDGFB, PLAU, TIMP3, TNC, TPM1
ECM-receptor interaction	8	81	6.8 x 10^{-3}	COL4A6, COL6A1, ITGA3, ITGA5, ITGA6, LAMA3, LAMC2, TNC
Human papillomavirus infection	15	317	2.4 x 10^{-2}	CCNA1, CCND1, CDK6, COL4A6, COL6A1, FADD, HES7, ITGA3, ITGA5, ITGA6, LAMA3, LAMC2, PPP2R2C, TNC, WNT7A
Cytokine-cytokine receptor interaction	13	263	3.1 x 10^{-2}	CCL27, CSF2, CXCL14, EPO, IFNK, IL11, IL24, INHBA, INHBB, PDGFB, PF4V1, TNFRSF12A, VEGFC
Gap junction	7	87	3.1 x 10^{-2}	ENSG00000258947, GJA1, GNAI1, PDGFB, TUBA4A, TUBB2A, TUBB6
Protein digestion and absorption	7	90	3.2 x 10^{-2}	COL13A1, COL4A6, COL5A3, COL6A1, DPP4, MME, SLC7A8
Small cell lung cancer	7	92	3.2 x 10^{-2}	CCND1, CDK6, COL4A6, ITGA3, ITGA6, LAMA3, LAMC2
Hematopoietic cell lineage	7	94	3.3 x 10^{-2}	CSF2, EPO, IL11, ITGA3, ITGA5, ITGA6, MME

Differentially expressed genes (DEGs); Kyoto Encyclopaedia of Genes and Genomes (KEGG).

Supplementary Material and methods

The amount of HPV16 mRNA was normalized to the quantity of GAPDH mRNA. Quantitative GAPDH-PCR was performed where each 25 µL PCR contained QuantiTect Probe RT-PCR Mastermix (Qiagen), 0.4 µM of each primer (position 595 forward 5′-AGCCTCAAGATCATCAGCAATG, position 691 reverse 5′-TGAGTCCTTCCACGATACCAAAGT) 0.05 µL M of the probe (position 647-662, FAM-TGGCCAAGGTCATCCA-MGB) (positions according to NM_001289745.2 available in GenBank) and 2.5 µL sample (extracted using the Oligotex Direct mRNA Mini Kit; Qiagen). Each sample was analysed in duplicate. PCR was performed on an Applied Biosystem Thermal Cycler 7500, as follows; 30 sec. at 50°C, 15 sec. at 95°C and then 45 cycles of 15 sec. at 95°C and 1 min. at 60°C. Quantity was extrapolated

from a linear regression standard curve obtained from serial dilutions of 2.5×10^6 to 2.5×10^3 copies per PCR of the above GAPDH-amplicon in a plasmid vector (PCR®2.1-TOPO®, Invitrogen).
